# Psychometric properties of the PROMIS-57 questionnaire, Norwegian version

**DOI:** 10.1007/s11136-021-02906-1

**Published:** 2021-06-18

**Authors:** Stein Arne Rimehaug, Aaron J. Kaat, Jan Egil Nordvik, Mari Klokkerud, Hilde Stendal Robinson

**Affiliations:** 1 University of Oslo, Oslo, Norway; 2 Sunnaas Rehabilitation Hospital, Bjørnemyr, Norway; 3 Northwestern University, Chicago, USA; 4 CatoSenteret Rehabilitation Center, Son, Norway

**Keywords:** PROMIS, Patient-reported outcomes, Quality of life, Clinimetric, Psychometric, Validity

## Abstract

**Purpose:**

The aims of this cross-sectional study were to explore reliability and validity of the Norwegian version of the Patient-Reported Outcome Measurement System^®^—Profile 57 (PROMIS-57) questionnaire in a general population sample, *n* = 408, and to examine Item Response properties and factor structure.

**Methods:**

Reliability measures were obtained from factor analysis and item response theory (IRT) methods. Correlations between PROMIS-57 and RAND-36-item health survey (RAND36) were examined for concurrent and discriminant validity. Factor structure and IRT assumptions were examined with factor analysis methods. IRT Item and model fit and graphic plots were inspected, and differential item functioning (DIF) for language, age, gender, and education level were examined.

**Results:**

PROMIS-57 demonstrated excellent reliability and satisfactory concurrent and discriminant validity. Factor structure of seven domains was supported. IRT assumptions were met for unidimensionality, local independence, monotonicity, and invariance with no DIF of consequence for language or age groups. Estimated common variance (ECV) per domain and confirmatory factor analysis (CFA) model fit supported unidimensionality for all seven domains. The GRM IRT Model demonstrates acceptable model fit.

**Conclusions:**

The psychometric properties and factor structure of Norwegian PROMIS-57 were satisfactory. Hence, the 57-item questionnaire along with PROMIS-29, and the corresponding 8 and 4 item short forms for physical function, anxiety, depression, fatigue, sleep disturbance, social participation ability and pain interference, are considered suitable for use in research and clinical care in Norwegian populations. Further studies on longitudinal reliability and sensitivity in patient populations and for Norwegian item calibration and/or reference scores are needed.

**Supplementary Information:**

The online version contains supplementary material available at 10.1007/s11136-021-02906-1.

## Plain language summary

PROMIS-57 is a questionnaire for self-reporting different aspects of physical, mental, and social health in adults. There are sections for physical function, anxiety, depression, fatigue, sleep problems, social participation and pain measurement. This study examined the Norwegian version by having 408 persons complete the questionnaires PROMIS-57 and RAND-36, another commonly used questionnaire, and testing the results with a variety of advanced statistical methods to see if PROMIS-57 is able to accurately measure these different components of a healthy life. The results indicate that this is the case, and that the translated questionnaire may be used in research and in health care to measure the results of treatment, or to measure the burden of living with a health condition or disability.

## Introduction

Patient-reported outcomes measurement (PROM) based on standardized questionnaires have become essential tools for health research and patient-centered care. The Patient-Reported Outcomes Measurement Information System^®^ (PROMIS) initiative has introduced flexible ways to select and develop PROM’s and provided new item banks, short form questionnaires, as well as flexible computerized adapted testing, that are growing in popularity among health researchers and in clinical services [1]. PROMIS item banks were developed via factor analysis and item response theory (IRT) [2, 3]. This measurement system encompasses several hundred items across many item banks, each covering a different physical, mental, or social health domain [2], as well as fixed short forms, and profiles such as PROMIS-57 and PROMIS-29. These PROMIS profiles have been described and validated in previous studies [1, 4–7].

Cultural and linguistic differences or translation issues may cause any translated questionnaire to have different psychometric properties than the original, so the properties of translated versions need to be examined. The objective of this study was to explore the reliability and validity of the Norwegian PROMIS-57 and -29 according to standards issued by the PROMIS Health Organization (PHO) [8] for validation after translation, and using RAND-36 as comparative reference. The short forms embedded in PROMIS-57 were hypothesized to have strong internal consistency, a strong concurrent and discriminant validity against RAND-36, satisfactory IRT properties, factor structure confirmed, no differential item functioning (DIF) for language, age, gender, education level, or self-reported health.

## Methods

This study was cross-sectional, and collection of responses was collected in a sample from the general population. Respondents were recruited in 2019 through a newspaper advertisement and posts on Facebook groups and pages encouraging sharing of a link to an online questionnaire. This questionnaire included a consent statement and information about the purpose of the study (Online appendix section S1).

### Measures

Participants filled in their responses to all items in the Norwegian PROMIS-57 and RAND-36, and the following demographic information was collected: gender, age, education level, employment status, income categories, cohabitation, and presence of mental and/or physical health concern.

PROMIS Profile 57 (PROMIS-57) is a collection of eight-item PROMIS short forms meant to capture important health domains. The following seven domains are included: physical function (PF), anxiety (ANX), depression (DEP), fatigue (FAT), sleep disturbance (SLP), ability to participate in social roles and activities (SOC), pain interference (PAIN), and a pain intensity numeric rating scale (NRS). The concepts and properties of these domains have been previously described [1, 9]. PROMIS-57 has recently been translated into Norwegian by the main author and colleagues, in cooperation with the Director of Translations for PROMIS, and approved according to rigid standards set forth by the PROMIS Health Organization [10]. This is the first study to examine the psychometric properties of the Norwegian version of PROMIS-57. Cultural bias from using US reference *T*-scores in Western Europe has previously been shown to be minimal [7, 11]. There is still a need to confirm that the psychometric properties (including monotonicity, unidimensionality, local independence, etc.) are supported in this translation, and to check DIF in this version.

PROMIS-29 is a shorter questionnaire nested within PROMIS-57, consisting of four items from each of the seven domains; thus the properties of PROMIS-29 can be examined using the same data.

Each item on the PROMIS-57 has 5 response options, except for the 0–10 pain intensity NRS item. Raw scores for each of the seven short forms (domains) in PROMIS-57 were calculated, and scores were converted to *T*-scores for each of the seven short forms (domains) in PROMIS-57 using the online scoring at www.assessmentcenter.net/ac_scoringservice [12] (Details in online appendix section S2). The *T*-score conversion establishes 50 as a general population mean for all PROMIS domains, and any 10-point deviation corresponds to one standard deviation (SD) difference, for easy-to-understand and consistent scoring across measures. Higher scores in any PROMIS scale indicate more of the measured construct, such that correlations between function and severity domains would be negative. *T*-scores, rather than raw scores, should always be used when applying PROMIS measures in clinical care and studies.

RAND-36-item Health Survey 1.0 (RAND-36) [13] reliability and validity are well established across diverse populations [14]. It is license and cost free, and covers eight life domains labeled as physical functioning (PF), bodily pain (BP), role limitations due to physical health problems (RP), role limitations due to personal or emotional problems (RE), general mental health (MH), social functioning (SF), energy/fatigue or vitality (VT), and general health perceptions (GH). RAND-36 contains the same items as the original version Short Form-36 (SF-36) [13], but has a different scoring system. The 3-, 5- or 6-category responses were converted to sum scores, using the official RAND-36 scoring syntax [14], so that higher scores indicate better health on a 0–100 scale for each of the eight domains.

### Statistical analyses

The methods chosen for analysis were based on the criteria in the COSMIN risk-of-bias checklist [15], the PROMIS Standards for release of PROMIS instruments after translation v.8 [8] and PROMIS Instrument Development and Validation Scientific Standards Version 2.0 [10]. These standards require an evaluation of reliability, validity, and assumption checking for IRT modeling. Ordinal item scores were used for factor analysis, IRT, and DIF analyses. Raw sum scores were used to explore floor/ceiling effects, while *T*-scores were used for correlations and for presenting mean *T*-scores per domain.

#### Reliability and internal consistency

We used reliability measures based on factor analysis and IRT, calculating marginal reliability and McDonald’s omega coefficients from an exploratory bi-factor analysis in R package ‘psych’ v1.8.12, expecting excellent reliability > 0.9 for each of the domains, as found in other studies [16–18]. Measuring overall consistency for PROMIS-57 is not appropriate, since it is a multidimensional questionnaire, with no total score calculation. IRT Test Information Function and scale standard error (SE) plots were visually inspected to evaluate the reliability of measurement across the range of possible responses for each domain [19]. In addition, Cronbach’s alpha was calculated.

#### Validity

Concurrent validity of PROMIS-57 *T*-scores per domain were tested against their corresponding RAND-36 sub-scales using Spearman rho correlation coefficients (*r*_s_), considering *r*_s_ ≥ 0.8 as very strong correlation, 0.8 > *r*_s_ ≥ 0.7 as strong, and 0.7 > *r*_s_ ≥ 0.6 as moderate correlation strength. Discriminant validity was assessed through correlations between dissimilar PROMIS domain scores and RAND-36 sub-scales, expecting for instance physical, social, and pain scores to have low to moderate correlations (*r*_s_ < 0.6) with mental measures.

Factor validity was examined using confirmatory factor analysis (CFA). A 7-factor correlated traits CFA was fit, examining PROMIS-57 overall. Then, items from each domain were fit to a one-factor CFA for the relative fit of a single-factor, consistent with the unidimensionality assumption required to proceed to IRT analyses.

#### Item response analysis

Consistent with the PROMIS development process, and the existing USA calibrations, all seven domains within PROMIS-57 were separately analyzed with the graded response model (GRM), using R package ‘mirt’ v1.31. We hypothesized that the GRM would provide adequate fit and appropriate model coefficients, since original measure in English successfully used the GRM. Given some unexpected results (see below) and as a sensitivity analyses to our anticipated model, we evaluated the appropriateness of the GRM, contrasting it with the Generalized Rating Scale (GRSM) and Rasch partial credit model to inform the selection of IRT model and interpretation of higher than expected IRT discrimination parameters.

Prior to IRT modeling, the statistical assumptions of unidimensionality, local independence, and monotonicity were evaluated. We sought an eigenvalue ratio > 4:1 as signs of unidimensionality, calculated using the ‘psych’ package in R. We used bi-factor analysis, also in ‘psych’, to extract explained common variance (ECV) and McDonald’s omega hierarchical, indicating what proportion of variation is explained by the general factor and general factor saturation, which should be ECV > 0.60 and omega > 0.70 [20]. Further, CFA was performed to test the factor structure for unidimensionality, as stated above. CFA was performed using R package ‘lavaan’ v6.05 with the weighted least square mean and variance (WLSMV) adjusted estimator. Model fit for the factor analysis and for the IRT models was assessed, looking for the lowest Bayesian information criteria (BIC), and root mean square error of approximation (RMSEA) < 0.06, Standardized Root Mean Square Residual (SRMSR) < 0.08, Comparative Fit Index (CFI) > 0.95 and Tucker-Lewis Index (TLI) > 0.95 as reference values [21]. Model fit for IRT was examined through M2 analysis (type C2 because of the sample size) performed in R with ‘mirt’ package [22]. Local dependency (LD) was examined based on the residuals from the CFA with WLSMV estimator in R package ‘lavaan’, flagging any item pair with > 0.2 residual correlation, as in PROMIS item bank development [3]. LD was also examined with the Chen and Thissen LD index [23] in R package ‘mirt’, considering standardized *χ*^2^ of > 0.3 as possible LD and > 1 as definite LD. Monotonicity was tested using Mokken scale in R package ‘mokken’ [24], expecting scalability coefficients (coef_h) > 0.3. IRT item fit was examined using ‘mirt’ [22], expecting no items with an *S − χ*^2^ *p* value of less than 0.001, which would be indicative of poor item fit. The *S − χ*^2^ statistic indicates whether each item meets expected response frequencies under the estimated IRT model [25]. Also, IRT plots from the GRM were created with ‘mirt’, including the item response function (IRF), item characteristic curves (ICC’s) and item information curves, and these were visually inspected.

In addition to assumption checking and fitting the initial IRT model, we also evaluated DIF as a potential threat to validity via biased scores for only some sub-population. DIF analysis was performed using R package ‘lordif’ v0.3-3 [26] with ordinal logistic regression models. First we conducted ordinal logistic regression without an anchor and the *χ*^2^ criterion to identify potential items with DIF. Then we followed up the analyses using as anchor the items not exhibiting DIF, with McFadden’s pseudo *R*^2^-change of ≥ 2% as a critical value, as suggested by the PHO [8]. The impact of DIF on item scores and total domain score was examined by inspecting ICCs and test characteristic curves (TCCs), as in previous studies on PROMIS translation validation studies [11, 27, 28]. Language DIF was performed by comparing the scores in this study against two available PROMIS datasets from US studies, the ‘PROMIS Profiles HUI data’ [29] and the ‘PROMIS 1 WAVE1’ [30], including only the respondents who had completed all items within any given short form. Age DIF in the Norwegian sample was studied by grouping respondents as younger (*n* = 206) and older (*n* = 202) around the median age (52). Gender DIF was examined with 310 female and 98 male respondents. Education level DIF compared college/university level education (*n* = 299) vs. those with high school or lower (*n* = 109). Health DIF groups consisted of respondents reporting having “no health problems” (*n* = 130) vs. physical problems, mental health problems, or both (*n* = 278).

## Results

A total of 408 complete and anonymous responses were collected and all were included in the analysis. Characteristics of respondents are presented in Table 1. Responses to PROMIS-57 were complete for every item, and all response categories were endorsed in each domain, although category “5” has only < 10 respondents in five of the DEP and three ANX items. (Histograms of all domain scores are presented in the supplementary online appendix figure S1.)

**Table 1:** Sample characteristics: demographic variables and health status (*n* = 408)

Age—mean (SD^a^/min-max)		52 (13/19–88)
*n* (%)		
Women		310 (76)
Living alone	Employed, part or full time	102 (25)
	Retired	215 (53)
	Permanent disability	57 (14)
	Sick leave, short or long term^b^	79 (19)
	Other^c^	42 (10)
Income level	Low ( < 350 k NOK)	15 (4)
	Middle (350 k–600k NOK)	124 (31)
	High ( > 600 k NOK)	183 (45)
Education	College level or higher	96 (24)
	Intermediate	298 (73)
	Elementary only ( > 10 year)	89 (22)
Health problems, self reported	Physical health problems	21 (5)
	Mental health problems	166 (41)
	Both physical and mental	18 (4)
	No health problems	94 (23)

^a^Standard deviation

^b^Away from work > 12 month duration, «arbeidsavklaringspenger»

^c^Homemaker, student, no response or marked as «other»

### Reliability

The 8-item short forms within PROMIS-57 all had high reliability indices in this Norwegian sample, with McDonald’s omega total between 0.91 and 0.99, and IRT marginal reliability scores between 0.87 and 0.94, and Cronbach’s alpha values between 0.91 and 0.98, see Table 2 for details. Floor/ceiling effects (% respondents at the max/min raw sum score) were quite high in this sample, especially for PF, see Table 2.

**Table 2:** PROMIS-57 per domain mean scores, and reliability and validity variables

PROMIS domain	Physical function	Anxiety	Depression	Fatigue	Sleep Dstrb	Social R&A	Pain Intf	Pain intensity NRS
Mean *T*-score (SD^a^)	47.6 (10.6)	50.8 (11.0)	51.3 (13.0)	52.3 (11.0)	52.6 (9.9)	48.3 (12.2)	55.0 (11.8)	3.5^b^ (2.8)
Ceiling%	36.4	.0	1.0	2.4	1.5	21.5	6.8	1.2
Floor%	0.5	24.2	29.6	17.6	2.7	3.9	29.1	19.6
Cronbach’s alpha	0.97	0.96	0.97	0.98	0.92	0.98	0.98	
McDonalds omega	0.97	0.96	0.97	0.98	0.92	0.99	0.99	
*ω*_*t*, (*ω*_*h*)^c^	(0.96)	(0.95)	(0.96)	(0.98)	(0.91)	(0.99)	(0.99)	
IRT Marginal reliability	0.87	0.91	0.89	0.94	0.92	0.93	0.90	
IRT discrimination mean^d^	5.9	4.7	4.8	7.4	4.0	7.5	8.5	
Eigenvalue ratio per domain^d^	12:1	10:1	16:1	33:1	5:1	54:1	26:1	
Explained common variance (ECV)^e^	0.88	0.86	0.88	0.96	0.74	0.96	0.94	

^a^Standard Deviation

^b^Pain NRS mean is not a *T*-score

^c^*ω*_*t* = omega total, *ω*_*h* = hierarchical

^d^IRT Discrimination parameter from Graded Response model. All confidence intervals (95%) for alpha and omega are <  ± .01, except Sleep: ± .02

^e^Obtained from bi-factor analysis with R package psych

**Fig. 1:**
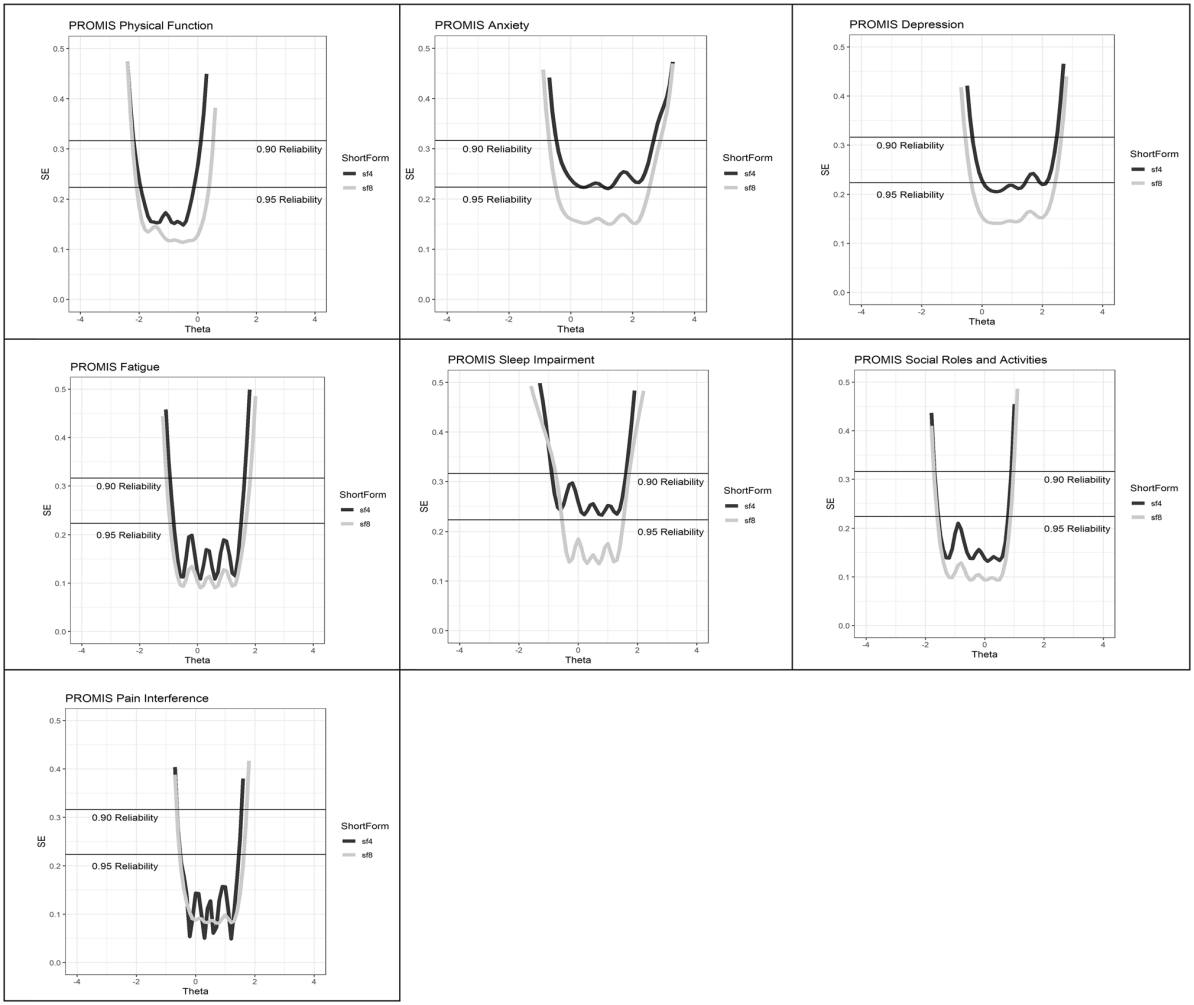
PROMIS-57 and 29 Standard error plots per domain, from Graded Response Model, reliability range. The horizontal axis represents the different ability/problem levels for each domain. *θ* = 0 representing the estimated mean from the IRT model, with a standard deviation of 1. The vertical axis represents the standard error (reliability), with reference reliabilities of .90 and .95. The lower the curve, the greater the reliability

Plots for the IRT standard error ranges in Fig. 1 were satisfactory, except for Sleep disturbance 8, where reliability was lower at both ends of the theta range. These plots show a small difference in reliability across the range. Both the 4- and 8-item short forms (associated with the PROMIS-29 and -57, respectively) were reliable within a range of the theta (the “ability” or “problem” range) that is relevant to health measurement, from about one SD better than the population average to at about two SD worse (below 0 for negatively scored PROMIS domains; anxiety, fatigue, pain).

### Validity

Strong correlations were found between comparable domains on the PROMIS and RAND-36. PROMIS Physical function and RAND-36 PF (0.88), social SF (0.89), and FAT and VT (-0.86), DEP and MH (− 0.81), ANX and MH (− 0.73), PAIN and BP (− 0.93), and between PROMIS Pain intensity NRS and RAND-36 BP (− 0.92). See details in Table 3.

**Table 3:** Spearman rho correlations *r*_s_ within PROMIS-57 domains and against RAND-36 subscores

PROMIS	Physical function	Anxiety	Depression	Fatigue	Sleep	Social^a^	Pain^b^	PainNRS^c^
Physical function	1.000	− 0.409	− 0.501	− 0.750	− 0.541	0.822	− 0.815	− 0.741
Anxiety	− 0.409	1.000	0.759	0.591	0.547	− 0.532	0.438	0.449
Depression	− 0.501	0.759	1.000	0.642	0.546	− 0.585	0.509	0.462
Fatigue	− 0.750	0.591	0.642	1.000	0.608	− 0.857	0.728	0.688
Sleep	− 0.541	0.547	0.546	0.608	1.000	− 0.593	0.547	0.533
Social^a^	0.822	− 0.532	− 0.585	− 0.857	− 0.593	1.000	− 0.774	− 0.691
Pain^b^	− 0.815	0.438	0.509	0.728	0.547	− 0.774	1.000	0.918
Pain^c^ NRS	− 0.741	0.449	0.462	0.688	0.533	− 0.691	0.918	1.000
RAND 36								
RAND36 PF PHYSICAL	0.880	− 0.329	− 0.422	− 0.675	− 0.513	0.751	− 0.781	− 0.731
RAND36 RP ROLEPHY	0.786	− 0.420	− 0.479	− 0.738	− 0.509	0.794	− 0.737	− 0.688
RAND36 BP BODILYPAIN	0.793	− 0.414	− 0.468	− 0.713	− 0.526	0.741	− 0.927	− 0.918
RAND36 GH GENERAL	0.776	− 0.524	− 0.558	− 0.776	− 0.620	0.785	− 0.718	− 0.681
RAND36 VT VITALIT	0.715	− 0.560	− 0.632	− 0.864	− 0.617	0.827	− 0.670	− 0.622
RAND36 SF SOCIAL	0.785	− 0.517	− 0.587	− 0.827	− 0.597	0.885	− 0.743	− 0.683
RAND36 RE ROLEMOT	0.389	− 0.545	− 0.584	− 0.524	− 0.441	0.488	− 0.417	− 0.432
RAND36 MH MENTAL	0.467	− 0.727	− 0.806	− 0.644	− 0.560	0.574	− 0.480	− 0.451

^a^Social roles and activities ability

^b^Pain interference

^c^Pain intensity numeric rating scale

PROMIS-57 discriminated well between physical and mental scores, as PROMIS anxiety and depression scores correlated only moderately (*r*_s_ < 0.5) with RAND-36 PF and RP, as well as between PROMIS Physical Function and RAND-36 RE and MH, and between PROMIS pain interference and RAND-36 RE and MH. The remaining correlations among PROMIS and RAND-36 dimensions were moderate to strong (*r*_s_.5–*r*_s_.8).

Weaker correlations were found, as expected, within PROMIS-57; *r*_s_ < 0.5 between PF/PAIN and ANX/DEP. Moderate correlation (*r*_s_ > 0.6) between SOC and ANX/DEP, between FAT and ANX, and between SLP and all other PROMIS dimensions. As expected, PF, FAT, SOC and PAIN were more closely related, with correlations well above *r*_s_ 0.7. Details in Table 3.

#### Unidimensionality (factor validity)

The correlated seven-factor CFA solution using WLSMV estimator for the entire PROMIS-57 produced a satisfactory model fit, confirming the original factor structure of seven domains within PROMIS-57. Scaled fit indices were CFI = 0.94, TLI = 0.94, RMSEA = 0.04, and SRMR = 0.04. The average absolute residual correlation was 0.002, and no residual correlations were > 0.2. From a single-factor CFA using WLSMV estimator performed *separately* for each domain, most scaled fit indices were within the acceptable thresholds, though less ideal for SLP than the other domains (Table 4). RMSEA criteria of > 0.06 were not met for any domain, but that is not uncommon for PROMIS and similar questionnaires [31].

**Table 4:** Single-factor CFA fit, all PROMIS-57domains tested separately with WLSMV estimator

	rmsea.scaled	srmr	cfi.scaled	tli.scaled
Physical function	0.129	**0.022**	**0.998**	**0.997**
Anxiety	0.080	**0.019**	**0.998**	**0.998**
Depression	0.124	**0.023**	**0.996**	**0.994**
Fatigue	0.115	**0.010**	**0.999**	**0.999**
Sleep disturbance	0.223	**0.074**	**0.986**	**0.980**
Social roles and activity	0.124	**0.011**	**0.999**	**0.999**
Pain interference	0.156	**0.015**	**0.999**	**0.999**
Commonly used Cutoffs	< 0.06	< 0.08	> 0.95	> 0.95

Bold = meets cutoff

### IRT analysis

Assumptions for IRT were satisfied for all seven short forms. Unidimensionality was supported by ECV from bifactor models between 0.74 and 0.96, omega hierarchical greater than 0.70, and ratio of first to second eigenvalues was greater than 4:1 for all domains. The factor structure with seven domains was supported by the CFA.

Each domain was considered locally independent, since no item pair residuals from the CFA are > 0.2 in any domain, and the standardized Chen and Thissen LD index for each domain flagged no pairs > 1, and only four pairs > 0.3; two FAT, one SLP, one SOC. (Details in supplementary online appendix Table S3.) Monotonicity was supported, as Mokken scalability coefficient for each domain scale was between 0.62 (SLP) and 0.93 (PAIN), well above the 0.3 cutoff, and no single item lower than 0.49 (Item Sleep116). (Details in the supplementary online appendix 1 Table S1).

PROMIS-57 had good IRT Item fit with GRM, except for two Sleep disturbance items with *S − χ*^2^ *p* values < 0.001, with or without FDR False Discovery Rate correction [32]. The misfitting items were Sleep44 and Sleep72.

**Fig.2:**
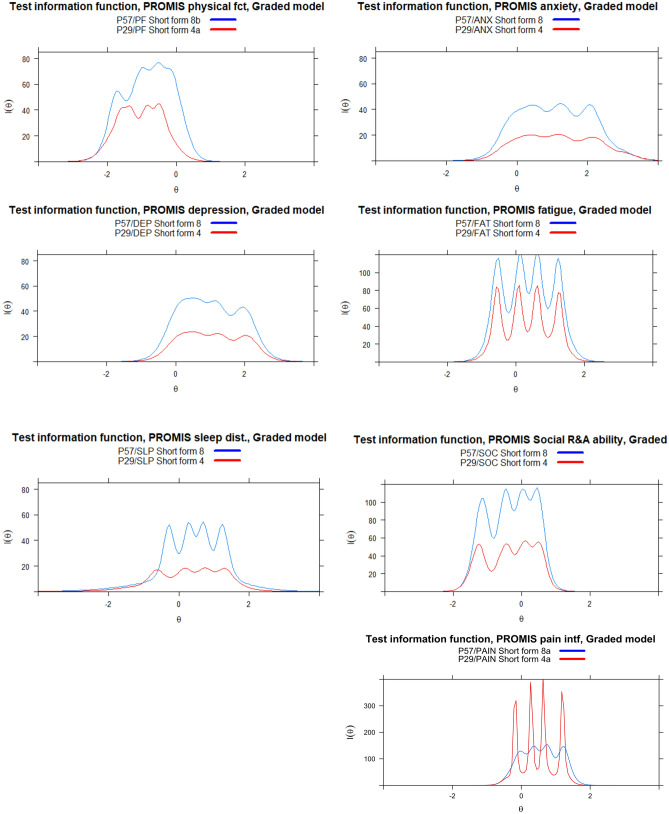
PROMIS-57 vs PROMIS-29 comparison of IRT test information function (TIF) plots. The horizontal axis represents the different ability/problem levels for each domain, with *θ* = 0 representing the estimated mean from the IRT model, with a standard deviation of 1. The vertical axis represents the combined amount of IRT information from all items of that particular scale

Item response curves generated in the ‘mirt’ package in R to visualize reliability displays well distributed curves, generally without response category curves completely overlapped by others, except item Sleep 116 and PF53 (Physical function). However, steep slopes for some items indicate high discrimination parameters, also evident as spiked Test Information curves (Fig. 2).

Comparing the test information function (TIF) of PROMIS-57 and PROMIS-29, the information precision is lower in the shorter versions (i.e., the PROMIS-29 or the included 4-item short forms). Some of the TIF curves appear spiked with the GRM, related to their high discrimination parameters. IRT parameters and plots for the individual items are available in supplementary online appendix Table S1. Given the steep slopes with the Norwegian calibrations, we conducted a sensitivity analyses to test the appropriateness of the GRM to the obtained data. Alternative models included the GRSM and the Rasch partial credit model. The GRM provided better fit than the Rasch model for all domains, but the GRSM provided better fit to Physical Function, Anxiety, DEP, Social and Pain. (Fit indices, see supplementary online appendix Table S2.)

### Differential item functioning

When applying suggested thresholds, no language, age, gender, education DIF of consequence was found. Along the way to this conclusion, however, there were some findings worth exploring.

**Fig. 3:**
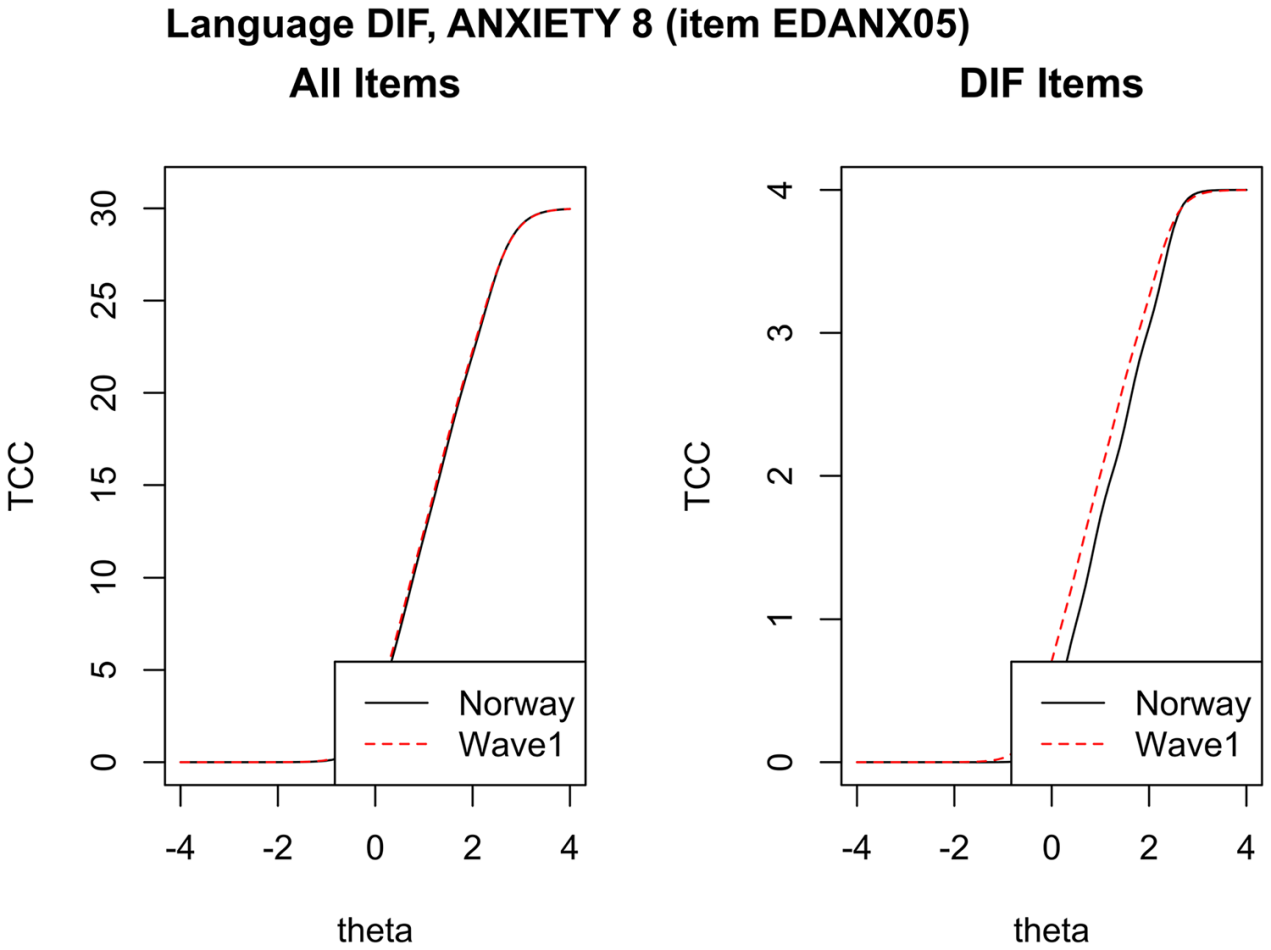
Test characteristic curves (TCC) for Norway/USA language DIF in PROMIS Anxiety 8 Short Form. Left graph shows the TCC total consequence of DIF on the scoring of all 8 Norwegian (Norway) and United States (Wave1) PROMIS Anxiety items; the right graph shows the TCC for just EDANX05 with negligible DIF

*Language DIF*: Three items in PROMIS-57 could not be tested for English vs Norwegian language DIF; item PFC12 as it was not included in either of the US reference data sets, and EDANX07 and Sleep72 as they were collected from other US respondents than the remaining items. Only respondents that had been presented with the same items in the same domain were selected from the US data sets, *n* = 1214 in Wave1 and *n* = 3409 in Profiles-HUI. DIF analysis with the over-sensitive chi-square (*χ*^2^) criterion, alpha threshold = 0.01, typically flagged one or more items per domain initially. Using the Δ*R*^2^ criterion suggested by the PHO (i.e., lordif settings: pseudo.R2 = ”McFadden”, criterion = ”R2”, R2.change = 0.02, model = ”GRM”), and using as anchors 2–3 DIF free items, as identified by the *χ*^2^ method [33], there was language DIF against the US datasets in only one item PAININ09 in all PROMIS-57 short forms. Running DIF analysis without anchors, language DIF was flagged for one item (but not flagged without anchors), EDANX05 against Wave1 dataset (Fig. 3). These same items were not flagged as DIF against the other US dataset (Profiles-HUI).

*Gender DIF*: There were some differences between gender mean scores, but no gender DIF detected in any of the seven PROMIS short forms in PROMIS-57. *Age DIF*: Three PROMIS-57 short forms (FAT, ANX, and PAIN) were free of DIF between older and younger respondents with either method. Uniform DIF was detected for one Physical Function item only with the *χ*^2^ method, but none with the pseudo R2 method. Depression: uniform DIF was detected for two items only with the *χ*^2^ method, but not with the R2 method. Two short forms, Sleep disturbance and Social roles showed non-uniform age DIF in one item only with the *χ*^2^ method, but not with the R2 method. *Education DIF*: No item in any short form was flagged for education DIF, comparing with/without college level. *Health status DIF*: unable to run for two short forms (PF and ANX) as some of the response categories had too few respondents in the healthier group. No health status DIF was found in the remaining short forms (DEP, FAT, SLP, SOC, or PAIN).

## Discussion

This was the first study to assess the psychometric properties of PROMIS profile and short forms, Norwegian version. PROMIS-57 and -29 and the embedded short forms displayed sufficient validity and reliability for use as a generic clinical measure of physical, mental, and social health in adults. The high reliability of the domains (omega total and hierarchical, empirical reliability, and Cronbach’s alphas > 0.9) support the excellent internal consistency and reliability for the Norwegian version of PROMIS-57, as in other PROMIS studies [1, 4–7]. Visual inspection of the IRT SE plots generated from Norwegian-specific calibrations provides further evidence of excellent reliability in the most relevant range for most patient populations, from about population mean to 2SD’s worse. PROMIS-29 and its 4-item short forms has similar reliability to PROMIS-57, but with a somewhat narrower precision range beyond 1.5 SD’s worse than the mean (Fig. 1). Correlations against RAND-36 support the concurrent and discriminatory validity of PROMIS-57. *T*-scores were used to demonstrate the validity of the currently recommended scoring method. Previous studies have also found correlations across PROMIS and RAND-36/SF36 between 0.66 and 0.91 for similar constructs [34–37] and between 0.30 and 0.61 for dissimilar ones [5, 38]. The floor/ceiling effects in this sample were considerable, so these short forms may be more appropriate for disease populations than this general population sample.

The Norwegian translation has retained the original seven-factor structure, and has not introduced significant language DIF bias or age DIF, and likely no gender or education DIF, though more research with larger sample sizes is necessary as several of our comparisons had group sample sizes less than the recommended minimum of 200.

Some items yield very high IRT discrimination slopes (especially FAT, SOC and PAIN). Some of the SLP items show item misfit. Possible explanations are local independence violations, skewed or zero-inflated scores, and the sample size, which may be inadequate for IRT analysis in the presence of a non-normal distribution. Simulation studies looking into sample size for IRT modeling accept *n* > 200, [39], but caution that this depends on a few other factors. Model complexity, and too few respondents endorsing some of the categories, can bias the parameters estimated from the model [40]. LD is not present in the domains and items with inflated discrimination. The sample may have too many “non-cases”, or zero-inflation, which may result in inflate slopes [41, 42]. A recent simulation study suggests 1.5 to 2 points increased bias of discrimination with zero-inflation [43]. IRT discrimination, LD and item fit needs to be examined in larger and more diverse samples, or else ignored as it is in 1PL and Rasch models. These issues prompted us to compare model fit for alternative models. We chose to use the GRM, consistent with the existing PROMIS measures in English and the PHO translation standards. However, these elevated slopes led us to consider alternative IRT models. For five of the seven domains, the GRSM provided superior fit to the GRM. The GRM model fit indices are approaching established criteria of RMSEA < 0.06, SRMSR < 0.08, CFI > 0.95 and TLI > 0.95 [21], (details in online supplement Table S2)*.* M2 fit analysis on PROMIS-57 as a whole, supports using GRM. Absolute adherence to cutoffs are not needed when assessing model fit indices [44]. The GRM has been recommended for PROMIS measures [8], and the sensitivity analysis supports its use in several cases. Future research should also consider whether an alternative statistical model would be appropriate for PROMIS item domains.

Two items showed minimal language DIF, however the amount of DIF found in these two items was small and probably of no consequence to the total score, judged by the visual representations. Given the lack of DIF and the utility of PROMIS calibrations used internationally (especially with precedent from translations into other European languages) [7, 11], we propose continuing to use the USA calibrations for the Norwegian sample. As evidenced in Table 2, this general population Norwegian sample has broadly consistent *T*-scores to the USA, and is comparable to other Western European score distributions [7].

### Strengths and limitations

A strength of this study has been the application of more advanced statistical methods, exposing the questionnaire to closer scrutiny. Assessing seven PROMIS short forms at once has its advantages, as it allows for better comparison between domains. We maintained the original PROMIS emphasis on unidimensional domains, though the PROMIS Profiles are amenable to other advanced statistical methods, such as multidimensional IRT. This is an important future direction which could be considered for multiple languages—not just the Norwegian translation. Validating multiple PROMIS short forms at once is the first step expanding these item banks into Norway, while validation of entire item banks would allow testing the PROMIS system for full theta range reliability, floor/ceiling effect, and full calibration of the scale in this new language. Our sample could be more representative, as there was more self-reported health problems compared to the Norwegian general population, and more participants with higher education. Thirty-two percent of the sample reports no health problems vs 73% in the HUNT study [45], 73% in the present study had college level education vs 33% in the general population [46]. The 4.7 year age difference between the genders is significant, whereas gender associations with living alone, income level or taking prescription medications are not. The sample is also somewhat gender skewed, not unlike many patient populations in Norway.

Norwegian version of the PROMIS-57 and PROMIS-29 and embedded short forms are sufficiently reliable and valid to be used in clinical care and research. Future studies should explore longitudinal reliability and responsiveness in patient populations, as well as IRT calibration in a larger Norwegian sample.

## Supplementary Information

Below is the link to the electronic supplementary material.

Supplementary file1 (PDF 1076 kb)

## Data Availability

May be made available upon reasonable request.

## Code availability

R code used available on request, standard analysis from packages mirt, lavaan, psych, Mokken, ggplot2 and semTools.
