# Supplementary material for: Psychometric properties of the PROMIS-57 questionnaire, Norwegian version
Source: Qual Life Res. 2021 Jun 18;31(1):269–80. doi: 10.1007/s11136-021-02906-1 (PMC8800876; doi:10.1007/s11136-021-02906-1)
Supplement: Supplementary file 1 — Supplementary file1 (PDF 1076 kb) [file 11136_2021_2906_MOESM1_ESM.pdf]

Online appendix - supplementary material to

Psychometric properties of the of PROMIS-57 questionnaire, Norwegian version.

Authors: Stein Arne Rimehaug, Aaron J Kaat, Jan Egil Nordvik, Mari Klokkerud, Hilde Stendal Robinson

Submitted to Quality of Life Research, September 2020, revised March 2021

1. Supplementary Section S1 – Consent statement
2. Supplementary Section S2 – Example T-score conversion table
3. Supplementary Figure S1 – Raw score histograms
4. Supplementary Figure S2 – IRT Item Characteristic plots (ICC)
5. Supplementary Table S1 – IRT parameters for the Norwegian calibrations
6. Supplementary Table S2 – Alternative IRT model fit indices
7. Supplementary Table S3: Local independence output

1. Supplementary **Section S1**: Consent statement for online respondents **Utprøvning av et nytt skjema for egenrapportert helse** (In Norwegian)

Regional kompetansetjeneste for rehabilitering, Helse Sør-Øst, ber nå personer med og uten alvorlige helsetilstander, om å delta i utprøvningen av et nytt, internasjonalt måleskjema for egenrapportert helse. Undersøkelsen er anonym, og de som ønsker å delta, samtykker til deltakelse ved å gjennomføre og sende inn undersøkelsen. Resultatene vil både kunne bli benyttet i forbedringsarbeid i helsetjenesten og til forskning. Ingen besvarelser vil kunne spores tilbake til enkeltpersoner, og vil dermed heller ikke kunne kobles mot andre helsedata eller persondata.

I undersøkelsen vil man oppleve at flere spørsmål er like. Dette skyldes at vi her sammenligner et nytt skjema med et annet. Vi ber derfor om tålmodighet til å besvare hele undersøkelsen, som består av rundt 100 spørsmål, og tar mellom 15-20 minutter å fullføre i sin helhet. Det er viktig at du gjennomfører hele undersøkelsen, om du ønsker å delta.

Vi ber om at du deltar med kun 1 besvarelse. Ønsker du det, kan lenken til undersøkelsen deles med andre. Siden dette er en anonym undersøkelse, bruker alle som gjennomfører undersøkelsen den samme nettlenken. Resultatet fra undersøkelsen vil bli oppsummert og gjort tilgjengelig på våre websider (www.sunnaas.no/rkr) i tillegg til publisering i internasjonale tidsskrift.

2. Supplementary **Section S2**: T-score online scoring method

Preferred method for scoring multiple individuals: Assessment center online scoring service at https://www.assessmentcenter.net/ac_scoringservice

Upload .csv file with multiple assessments and receive accurate IRT theta.
T-score and Standard Error of measurement for each individual score by e-mail from AssessmentCenter.

Outside of the USA, make sure the .csv file is comma separated, and with periods for decimals, and later use the text-to-column function in Excel (or import functions in your statistics software like R or SPSS) to re-convert output files.

Alternatively, look up subscore conversion with the help of look-up tables and the PROMIS_Adult_Profile_Scoring_Manual.pdf found at www.healthmeasures.net

Refer to the resources for interpretation of scores at http://www.healthmeasures.net/score-and-interpret/

3. Supplementary **Figure S1** –HISTOGRAMS of PROMIS-57 SCORE distributions

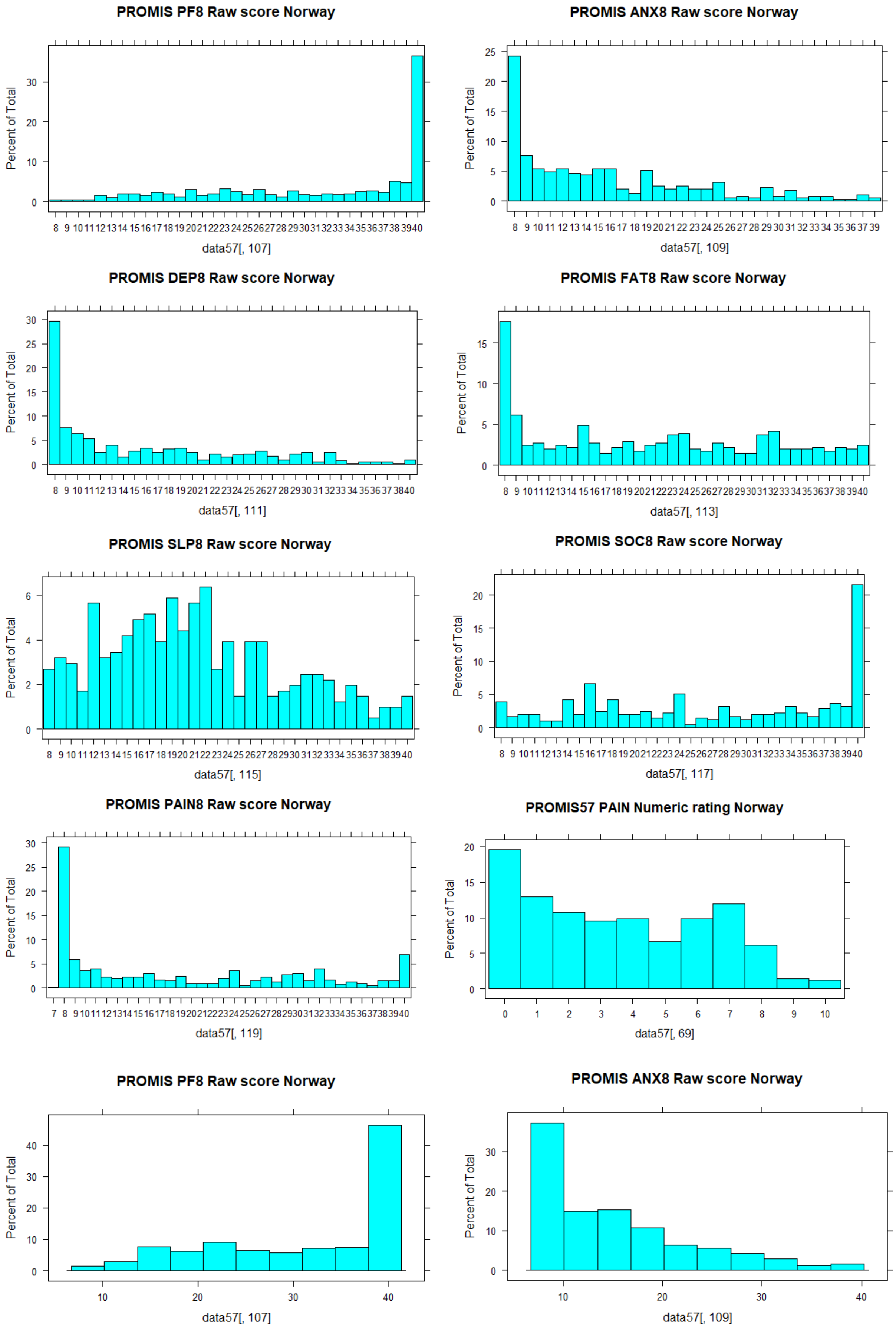

PROMIS DEP8 Raw score Norway

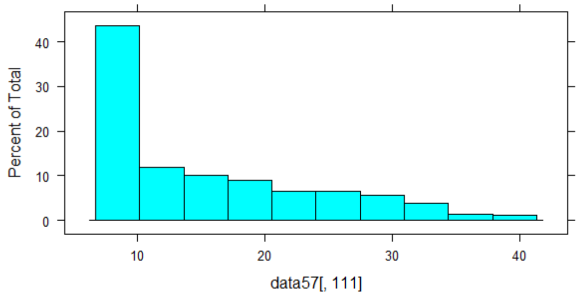

PROMIS FAT8 Raw score Norway

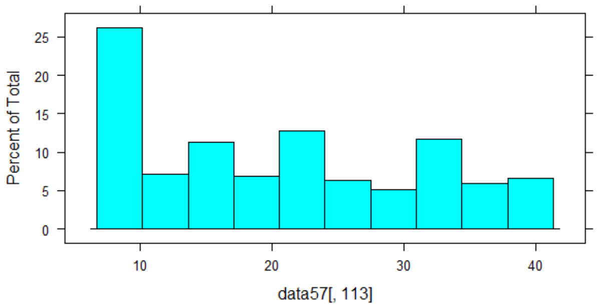

PROMIS SLP8 T-score Norway

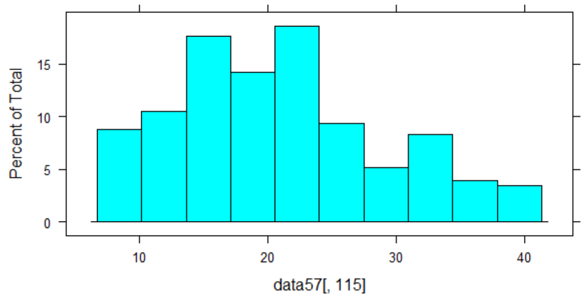

PROMIS SOC8 Raw score Norway

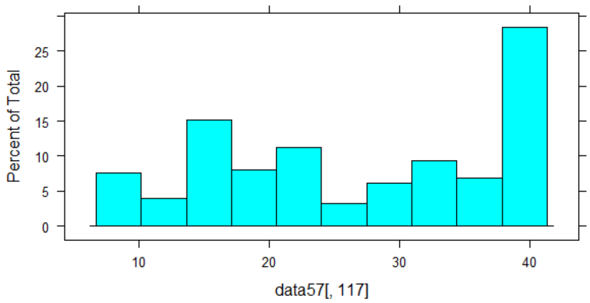

PROMIS PAIN8 Raw score Norway

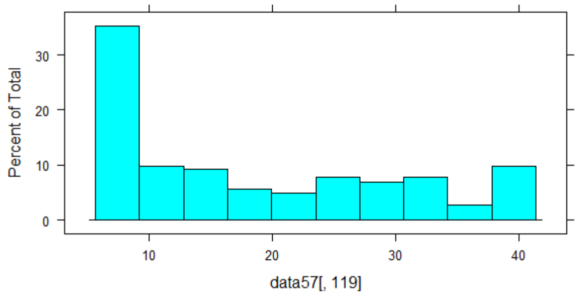

PROMIS57 PAIN Numeric rating Norway

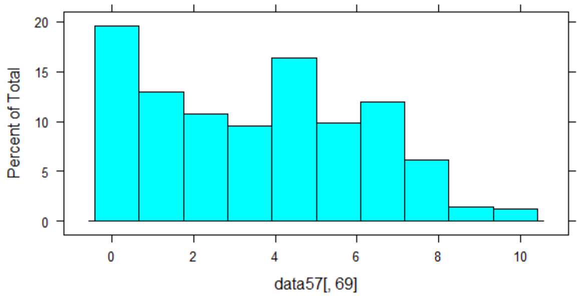

4. Supplementary **Figure S2** – IRT Item Characteristic plots (ICC)

Physical function IRT Item characteristic curves

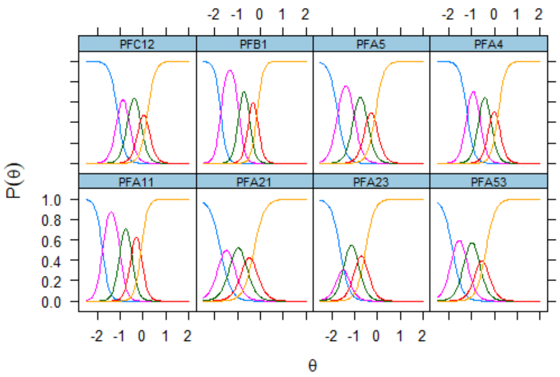

PROMIS Anxiety 8a IRT trace lines

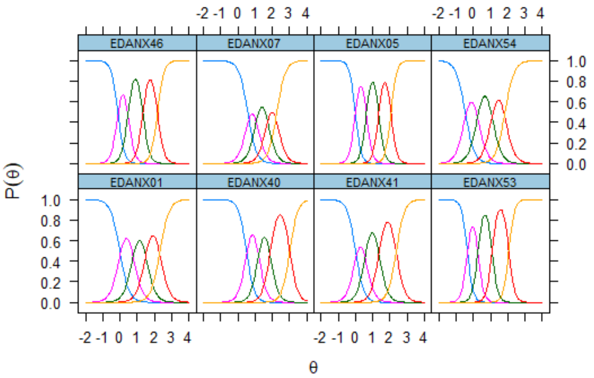

PROMIS Depression 8b Item trace lines

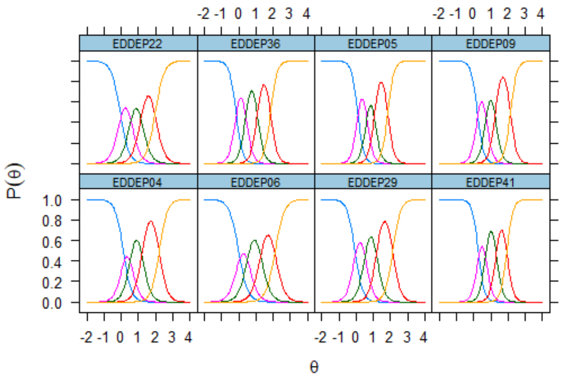

PROMIS Fatigue 8a IRT trace lines

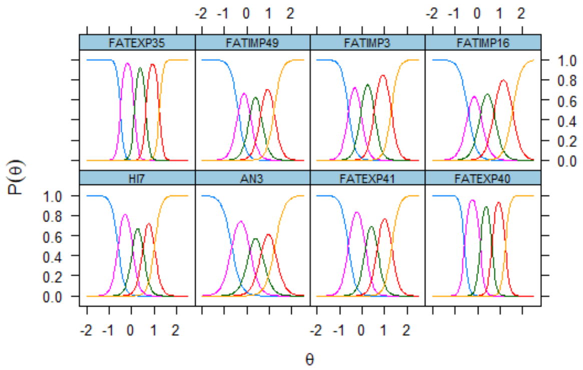

PROMIS Sleep dist 8b IRT trace lines

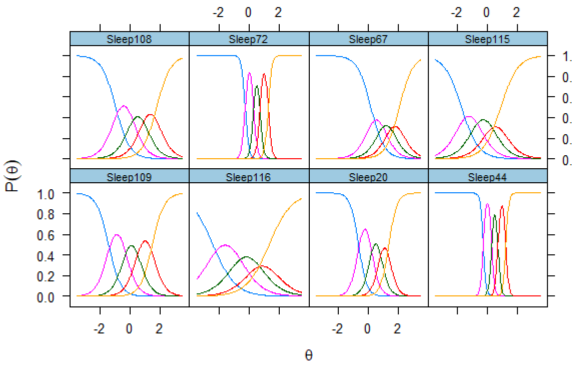

PROMIS Social R&A ability 8a IRT trace lines

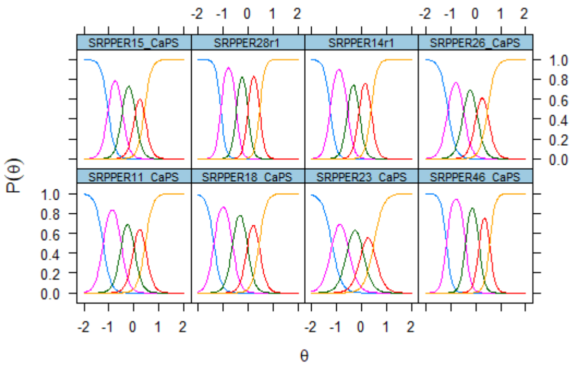

PROMIS Pain intf 8a IRT trace lines

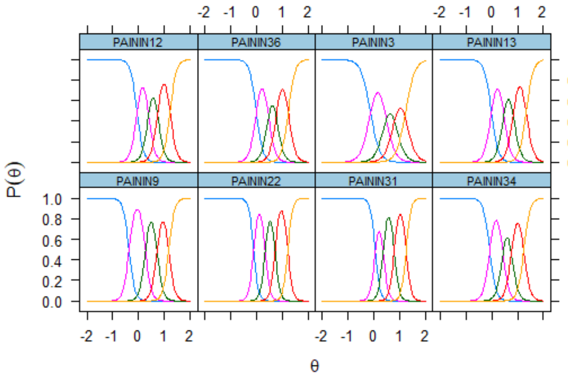

5. Supplementary **Table S1**: IRT parameters for the Norwegian calibrations

Physical function:	Scalability	a1	d1	d2	d3	d4	Average difficulty
PFA11	.885	7.001	-1.78	-1.01	-.51	-.09	-0.85
PFA21	.839	4.314	-1.73	-1.23	-.69	-.27	-0.98
PFA23	.861	5.078	-1.63	-1.38	-.89	-.52	-1.10
PFA53	.854	4.826	-1.83	-1.26	-.72	-.38	-1.05
PFC12	.891	6.072	-1.12	-.64	-.14	.20	-0.43
PFB1	.891	7.771	-1.73	-.93	-.49	-.13	-0.82
PFA5	.871	5.559	-1.74	-1.02	-.47	-.08	-0.83
PFA4	.890	6.421	-1.21	-.66	-.19	.16	-0.47
**Overall Scalability / Average Slope**	**.874**	**5.9**				**Avg of avg**	**-.82**

Anxiety:	Scalability	a1	d1	d2	d3	d4	Average difficulty
EDANX01	.814	3.76	-.01	.77	1.50	2.33	1.15
EDANX40	.841	4.393	.50	1.21	1.89	3.02	1.66
EDANX41	.817	4.157	.00	.58	1.37	2.37	1.08
EDANX53	.872	6.053	-.35	.27	1.09	2.06	0.77
EDANX46	.848	5.347	-.13	.47	1.33	2.18	0.96
EDANX07	.826	3.975	.58	1.10	1.72	2.27	1.42
EDANX05	.857	6.018	-.02	.63	1.35	2.06	1.00
EDANX54	.827	3.767	-.47	.25	1.08	1.84	0.68
**Overall Scalability / Average Slope**	**.838**	**4.7**				**Avg of avg**	**1.09**

Depression:	Scalability	a1	d1	d2	d3	d4	Average difficulty
EDDEP04	.816	4.239	.12	.57	1.22	2.22	1.04
EDDEP06	.807	3.737	.00	.55	1.29	2.12	0.99
EDDEP29	.813	4.456	-.07	.52	1.19	2.13	0.94
EDDEP41	.848	5.556	.27	.70	1.31	1.93	1.05
EDDEP22	.806	3.889	-.06	.57	1.18	1.99	0.92
EDDEP36	.851	5.25	-.16	.42	1.09	1.86	0.80
EDDEP05	.841	5.594	.09	.61	1.07	1.84	0.90
EDDEP09	.838	5.446	.21	.72	1.25	2.14	1.08
**Overall Scalability / Average Slope**	**.828**	**4.8**				**Avg of avg**	**.97**

Fatigue:	Scalability	a1	d1	d2	d3	d4	Average difficulty
HI7	.919	7.01	-.61	.04	.51	1.02	0.24
AN3	.874	4.786	-.68	.12	.66	1.25	0.34
FATEXP41	.897	6.331	-.62	.14	.68	1.32	0.38
FATEXP40	.919	1.803	-.58	.12	.64	1.24	0.35
FATEXP35	.926	12.51	-.50	.13	.63	1.24	0.38
FATIMP49	.899	5.987	-.39	.14	.63	1.21	0.40
FATIMP3	.917	6.564	-.61	-.06	.54	1.30	0.29
FATIMP16	.899	5.279	-.43	.14	.74	1.56	0.50
**Overall Scalability / Average Slope**	**.906**	**7.4**				**Avg of avg**	**.36**

Sleep interference:	Scalability	a1	d1	d2	d3	d4	Average difficulty
Sleep109	.686	2.501	-1.414	-.311	.546	1.504	0.08
Sleep116	.504	1.323	-2.42	-.781	.43	1.339	-0.36
Sleep20	.710	3.636	-.655	.198	.812	1.37	0.43
Sleep44	.697	1.211	-.291	.273	.693	1.222	0.47
Sleep108	.625	2.132	-.939	.122	.934	1.801	0.48
Sleep72	.694	8.391	-.262	.307	.725	1.282	0.51
Sleep67	.591	2.212	.175	.889	1.493	2.081	1.16
Sleep115	.569	1.763	-1.741	-.745	.159	.885	-0.36
**Overall Scalability / Average Slope**	**.637**	**4.0**				**Avg of avg**	**.30**

Social:	Scalability	a1	d1	d2	d3	d4	Average difficulty
SRPPER11_CaPS	.910	6.622	-1.24	-.51	.00	.46	-0.32
SRPPER18_CaPS	.919	7.017	-1.36	-.62	-.02	.45	-0.39
SRPPER23_CaPS	.891	5.244	-1.19	-.55	.02	.49	-0.31
SRPPER46_CaPS	.938	9.2	-1.17	-.40	.14	.56	-0.22
SRPPER15_CaPS	.914	7.308	-1.04	-.46	.05	.42	-0.26
SRPPER28r1	.927	1.023	-1.08	-.46	.00	.48	-0.27
SRPPER14r1	.930	8.302	-1.27	-.56	-.10	.38	-0.39
SRPPER26_CaPS	.910	6.472	-1.11	-.48	.04	.48	-0.27
**Overall Scalability / Average Slope**	**.917**	**7.5**				**Avg of avg**	**-.30**

Pain interference:	Scalability	a1	d1	d2	d3	d4	Average difficulty
PAININ9	.942	8.926	-.37	.26	.72	1.17	0.45
PAININ22	.937	1.84	-.11	.35	.73	1.22	0.55
PAININ31	.937	1.624	.05	.36	.78	1.25	0.61
PAININ34	.920	8.302	-.08	.43	.77	1.25	0.59
PAININ12	.928	8.234	-.06	.38	.74	1.22	0.57
PAININ36	.924	7.732	.00	.46	.78	1.25	0.62
PAININ3	.900	5.576	-.15	.44	.81	1.23	0.58
PAININ13	.930	7.488	-.02	.46	.84	1.34	0.66
**Overall Scalability / Average Slope**	**.927**	**8.5**				**Avg of avg**	**.58**

Scalability= coefH from Mokken analysis (R package Mokken) as an expression of monotonicity.

a1 = IRT discrimination (slope) parameter from GRM (R package mirt). d1-d4 = thresholds

IRT R-code example (Physical fct=PF): PFgrmodel <- mirt (PFdata57, 1, rep("graded", 8), SE = TRUE) coef(PFgrmodel, IRTpars=TRUE, simplify =TRUE)	Mokken package R code: coefH(PFdata57,se = TRUE, nice.output = TRUE, group.var = NULL, fixed.itemstep.order = NULL)

6. Supplementary **Table S2**: Alternative IRT model fit indices

Table S2: PROMIS-57, comparing five model fit indices[abcde] for three different IRT models:

Rasch Partial Credit Model (Rasch)/ Graded Response Model(GRM) / Generalized Rating Scale(GRSM), for each of the seven sub-domains, n=408

		PROMIS 57 domains/short forms:						
Fit indices, thresholds:		Physical Fct	Anxiety	Depression	Fatigue	Sleep	Social	Pain
BIC[a]<br>(lowest=best)	Rasch<br>GRM<br>GRSM	5200 /<br>5108 /<br>**5068**	5352 /<br>5258 /<br>**5202**	5536 /<br>5447 /<br>**5350**	5838 /<br>**5500** /<br>5501	8057 /<br>**7731** /<br>7781	5674 /<br>5367 /<br>**5299**	4863 /<br>5220 /<br>**4824**
RMSEA[b]<br><.06	Rasch<br>GRM<br>GRSM	.107 /<br>.115 /<br>.116	.091 /<br>.082 /<br>.076	.095 /<br>.098 /<br>.086	.138 /<br>.103 /<br>.106	.209 /<br>.227 /<br>168	.136 /<br>.116 /<br>.095	.145 /<br>.186 /<br>.138
SRMSR[c]<br><.08	Rasch<br>GRM<br>GRSM	.086 /<br>**.027 /**<br>**.040**	.092 /<br>**.025 /**<br>**.034**	**.075 /**<br>**.029 /**<br>**.030**	**.012 /**<br>**.013 /**<br>**.025**	.123 /<br>.081 /<br>.103	.120 /<br>**.013** /<br>**.020**	.119 /<br>**018 /**<br>**.027**
TLI[d]<br>>.95	Rasch<br>GRM<br>GRSM	**.098 /**<br>**.098 /**<br>**.097**	**.099 /**<br>**.099 /**<br>**.099**	**.098 /**<br>**.099 /**<br>**.099**	**.098 /**<br>**.099 /**<br>**.099**	.877 /<br>.856 /<br>.921	**.978 /**<br>**.983 /**<br>**.989**	**.974 /**<br>**.958 /**<br>**.977**
CFI[e]<br>>.95	Rasch<br>GRM<br>GRSM	**.098 /**<br>**.098 /**<br>**.097**	**.099 /**<br>**.099 /**<br>**.099**	**.099 /**<br>**.099 /**<br>**.098**	**.098 /**<br>**.099 /**<br>**.098**	.882 /<br>.897 /<br>.884	**.978 /**<br>**.988 /**<br>**.984**	**.975 /**<br>**.970 /**<br>**.966**
# of criteria met, for each model:	Rasch<br>GRM<br>GRSM	2/<br>3/<br>4	2 /<br>3 /<br>4	3 /<br>3 /<br>4	3 /<br>4 /<br>3	0 /<br>1 /<br>0	2 /<br>3 /<br>4	2 /<br>3 /<br>5

[a]Bayesian Information Criteria (BIC), [b]Root Mean Square Error of Approximation (RMSEA), [c]Standardized Root Mean Square Residual (SRMSR), [d]Comparative Fit Index (CFI) [e]Tucker-Lewis Index (TLI). [f]Rasch Partial Credit Model / [g]Graded Response / [h]Generalized Rating Scale. **Bold values** = criteria (thresholds) are met.

7. Supplementary Table S3: Local independence output

Table S3a: LD index from CFA with WLSMV estimator for PROMIS Sleep Disturbance 8

	Sleep109	Sleep116	Sleep20	Sleep44	Sleep108	Sleep72	Sleep67	Sleep115
Sleep109	.000							
Sleep116	**.105**	.000						
Sleep20	.040	-.061	.000					
Sleep44	-.107	-.164	-.035	.000				
Sleep108	.038	-.016	.084	-.105	.000			
Sleep72	-.121	-.152	-.060	.010	-.135	.000		
Sleep67	-.047	-.084	-.007	.007	.013	.039	.000	
Sleep115	.085	**.169**	-.006	-.127	-.023	-.124	-.012	.000

NONE with >.2 in any PROMIS57 domain. Two items >.1 in SLP (**bold** above), none in the other six domains.

Table S3b: IRT Residuals as expression of local dependence, output from R
LD matrix (lower triangle) and standardized residuals (upper triangle), all PROMIS 57 domains
Values >.3 are in **bold:**
(fair amount of LD, ref #23, Chen& Thissen 1997)

	PFA11	PFA21	PFA23	PFA53	PFC12	PFB1	PFA5	PFA4
PFA11	NA	.175	-.145	-.135	-.149	.261	.161	.173
PFA21	49.818	NA	.192	.141	-.095	-.185	-.166	-.169
PFA23	-34.259	6.101	NA	.187	.188	-.157	-.170	-.239
PFA53	-29.633	32.356	57.091	NA	.156	-.211	-.124	-.151
PFC12	-36.215	-14.795	57.472	39.585	NA	.148	.241	.140
PFB1	11.979	-55.860	-4.168	-72.785	35.899	NA	.124	.139
PFA5	42.206	-45.039	-47.331	-24.996	94.732	25.043	NA	.128
PFA4	48.814	-46.632	-92.943	-37.337	31.818	31.459	26.649	NA

	EDANX01	EDANX40	EDANX41	EDANX53	EDANX46	EDANX07	EDANX05	EDANX54
EDANX01	NA	.101	-.082	-.091	-.118	.173	.083	-.109
EDANX40	16.691	NA	.083	-.128	-.085	.131	-.107	-.105
EDANX41	-11.021	11.237	NA	.122	-.123	-.096	-.120	-.090
EDANX53	-13.469	-26.856	24.446	NA	-.122	-.106	-.138	.093
EDANX46	-22.777	-11.801	-24.544	-24.381	NA	.109	-.172	.100
EDANX07	48.638	27.858	-14.916	-18.222	19.354	NA	.137	-.119
EDANX05	11.264	-18.860	-23.444	-31.300	-48.464	3.618	NA	-.152
EDANX54	-19.529	-18.138	-13.240	14.071	16.428	-23.098	-37.769	NA

	EDDEP04	EDDEP06	EDDEP29	EDDEP41	EDDEP22	EDDEP36	EDDEP05	EDDEP09
EDDEP04	NA	.193	.265	-.186	.147	-.162	-.252	-.202
EDDEP06	6.801	NA	.161	.095	.102	-.137	-.144	-.120
EDDEP29	114.346	42.238	NA	.146	-.152	.220	-.099	.117
EDDEP41	-56.552	14.814	34.916	NA	.122	-.081	-.093	-.098
EDDEP22	35.064	17.092	-37.621	24.438	NA	.197	-.159	-.142
EDDEP36	-42.939	-3.669	78.947	-1.580	63.180	NA	-.227	.079

EDDEP05	-104.001	-33.623	-16.058	-14.162	-41.010	-83.826	NA	.179
EDDEP09	-66.735	-23.478	22.323	-15.711	-33.017	1.150	52.459	NA

	HI7	AN3	FATEXP41	FATEXP40	FATEXP35	FATIMP49	FATIMP3	FATIMP16
HI7	NA	**.383**	-.404	.137	-.148	-.120	.095	.092
AN3	239.607	NA	-.708	-1.061	-1.134	-.485	-.379	**.437**
FATEXP41	-266.794	-818.496	NA	.264	-.123	-.118	-.750	-.221
FATEXP40	3.572	-1837.949	113.418	NA	.097	-.181	-.096	-.224
FATEXP35	-35.518	-2097.128	-24.688	15.421	NA	.120	-.203	-.215
FATIMP49	-23.455	-384.505	-22.622	-53.593	23.539	NA	.123	.174
FATIMP3	14.785	-234.731	-919.032	-14.990	-67.327	24.780	NA	.198
FATIMP16	13.780	312.307	-79.371	-81.922	-75.552	49.475	64.062	NA

	Sleep109	Sleep116	Sleep20	Sleep44	Sleep108	Sleep72	Sleep67	Sleep115
Sleep109	NA	.193	.234	-.306	.210	-.331	-.256	**.308**
Sleep116	6.986	NA	-.181	-.207	.147	-.172	-.145	.253
Sleep20	89.415	-53.172	NA	-.159	.205	-.215	-.127	-.280
Sleep44	-152.896	-7.107	-41.262	NA	-.212	.213	-.150	-.468
Sleep108	71.837	35.261	68.308	-73.194	NA	-.223	-.096	.299
Sleep72	-178.555	-48.376	-75.268	73.987	-81.242	NA	-.131	-.370
Sleep67	-106.728	-34.194	-26.427	-36.825	-15.005	-27.803	NA	-.256
Sleep115	155.123	104.277	-128.389	-357.357	145.771	-223.384	-107.263	NA

	SRPPER11_CaPS	SRPPER18_CaPS	SRPPER23_CaPS	SRPPER46_CaPS	SRPPER15_CaPS	SRPPER28r1	SRPPER14r1	SRPPER26_CaPS
SRPPER11_CaPS	NA	.115	-.114	.110	-.520	-.122	-.105	-.142
SRPPER18_CaPS	21.719	NA	.111	.111	-.149	-.101	.108	-.141
SRPPER23_CaPS	-21.385	2.054	NA	.131	-.112	-.117	-.120	.131
SRPPER46_CaPS	19.627	2.222	27.987	NA	.123	-.099	-.104	-.129
SRPPER15_CaPS	-441.035	-36.393	-2.625	24.533	NA	**.917**	-1.209	-.138
SRPPER28r1	-24.376	-16.637	-22.429	-16.027	1371.257	NA	.123	-.131
SRPPER14r1	-18.108	19.177	-23.665	-17.682	-2383.801	24.779	NA	.191
SRPPER26_CaPS	-33.133	-32.376	28.220	-27.070	-31.210	-28.152	59.710	NA

	PAININ9	PAININ22	PAININ31	PAININ34	PAININ12	PAININ36	PAININ3	PAININ13
PAININ9	NA	.116	.117	.112	-.107	-.255	-.112	-.143
PAININ22	22.052	NA	-.142	.159	-.143	-.173	-.155	-.140
PAININ31	22.328	-32.951	NA	-.135	.159	.106	-.165	-.139
PAININ34	2.296	41.027	-29.561	NA	-.170	-.148	-.213	-.123
PAININ12	-18.528	-33.152	41.300	-47.094	NA	.151	.134	.123
PAININ36	-106.454	-49.060	18.311	-35.696	37.248	NA	.128	.121
PAININ3	-2.579	-39.387	-44.453	-73.970	29.369	26.633	NA	.192
PAININ13	-33.549	-31.898	-31.557	-24.640	24.686	24.008	6.094	NA

LD RESULTS for Norwegian PROMIS PROFILE 57

Using CFA LD index: none at > .2 threshold (Reeve 2007)

Using IRT residuals (Chen&Thissen): 4 identified item pairs
FATIGUE: 2 pairs, SLEEP DISTURBANCE: 1 pair, SOCIAL ROLES AND ACT: 1 pair
= in total 4 pairs flagged for having LD >.3, out of 196 possible = only 2% LD

ItemID (standardized residuals) for the four flagged pairs:
Fatigue: HI7-AN3 (0.383), FATIMP16-AN3 (0.437)
Sleep: Sleep109-Sleep115 (0.325)
Social: SRPPER28r1-SRPPER15_CaPS (0.917)

R code example, PF=Physical Fct:
```
modPF <- mirt(PFdata57, 1, rep("graded", 8), SE = TRUE)
residuals(modPF, type = 'LD')
```
